# Alpha-1 antitrypsin inhibits *Clostridium botulinum* C2 toxin, *Corynebacterium diphtheriae* diphtheria toxin and *B. anthracis* fusion toxin

**DOI:** 10.1038/s41598-024-71706-7

**Published:** 2024-09-11

**Authors:** Stefanie Lietz, Lena-Marie Sokolowski, Holger Barth, Katharina Ernst

**Affiliations:** Institute of Experimental and Clinical Pharmacology, Toxicology and Pharmacology of Natural Products, Ulm University Medical Center, 89081 Ulm, Germany

**Keywords:** α_1_-Antitrypsin, Toxin inhibitor, *Clostridium botulinum* C2 toxin, Diphtheria toxin, Anthrax toxin, Drug repurposing, Bacterial infection, Bacterial infection

## Abstract

The bacterium *Clostridium botulinum*, well-known for producing botulinum neurotoxins, which cause the severe paralytic illness known as botulism, produces C2 toxin, a binary AB-toxin with ADP-ribosyltranferase activity. C2 toxin possesses two separate protein components, an enzymatically active A-component C2I and the binding and translocation B-component C2II. After proteolytic activation of C2II to C2IIa, the heptameric structure binds C2I and is taken up via receptor-mediated endocytosis into the target cells. Due to acidification of endosomes, the C2IIa/C2I complex undergoes conformational changes and consequently C2IIa forms a pore into the endosomal membrane and C2I can translocate into the cytoplasm, where it ADP-ribosylates G-actin, a key component of the cytoskeleton. This modification disrupts the actin cytoskeleton, resulting in the collapse of cytoskeleton and ultimately cell death. Here, we show that the serine-protease inhibitor α_1_-antitrypsin (α_1_AT) which we identified previously from a hemofiltrate library screen for PT from *Bordetella pertussis* is a multitoxin inhibitor. α_1_AT inhibits intoxication of cells with C2 toxin via inhibition of binding to cells and inhibition of enzyme activity of C2I. Moreover, diphtheria toxin and an anthrax fusion toxin are inhibited by α_1_AT. Since α_1_AT is commercially available as a drug for treatment of the α_1_AT deficiency, it could be repurposed for treatment of toxin-mediated diseases.

## Introduction

*Clostridium botulinum* C2 toxin is a binary AB-toxin produced by the bacterium *Clostridium botulinum*^1^. This bacterium is well-known for producing botulinum neurotoxins, which cause the severe paralytic illness known as botulism^1^. However, the C2 toxin is distinct from these neurotoxins, as it operates through a different mechanism and affects different cellular targets. The C2 toxin is composed of two separate protein components: the A-component C2I and the B-component C2II^2–4^. The C2I component has ADP-ribosyltransferase activity, meaning it can modify other proteins by adding ADP-ribose molecules^5^. The C2II component is responsible for binding to the surface of host cells and facilitating the translocation of the C2I component into these cells^6^. The mechanism of action for the C2 toxin involves several steps. The C2II component binds to specific receptors on the surface of target cells^7^. C2II undergoes proteolytic activation resulting in C2IIa^8^, which allows it to form a heptameric structure that can bind the C2I component^9^. The C2IIa/C2I complex is taken up by the cell via receptor-mediated endocytosis^10^. Acidification of endosomes leads to conformational changes in both components^11^. The heptameric C2IIa forms a pore into the endosomal membrane, and C2I is at least partially unfolded and translocates through the C2IIa pore into the cytoplasm^12–14^. This translocation process is supported by host cell chaperones like Hsp90, Hsp70, and peptidyl cis/trans isomerases^15^. Once inside the host cell, the C2I component exhibits its enzymatic activity by ADP-ribosylating G-actin, a key component of the cytoskeleton^5,16,17^. This modification disrupts the actin cytoskeleton, preventing actin polymerization, which is essential for maintaining the cell’s structure and shape. As a result, the cytoskeleton collapses, leading to cell rounding, loss of adhesion, and ultimately cell death^16^.

Diphtheria toxin (DT), produced by *Corynebacterium diphtheriae*, is a potent AB-toxin which harbors its A- and B-domain on one protein^18,19^. Uptake of DT is also facilitated by cell binding via the B-domain, receptor-mediated endocytosis, and translocation of the A-domain DTA from acidified endosomes to the cytosol^20–22^. Here, DTA ADP-ribosylates elongation factor 2 (EF-2), halting protein synthesis and causing cell death^23^. Clinically, the toxin leads to local tissue destruction and pseudomembrane formation in the throat, potentially obstructing the airway^18^. If it enters the bloodstream, it can cause myocarditis, neuropathy, and other systemic effects, which can be fatal^24^. Treatment includes early administration of diphtheria antitoxin to neutralize the toxin, antibiotics like penicillin or erythromycin to eliminate the infection, and supportive care^25^. Vaccination with the diphtheria vaccine, often combined with tetanus and pertussis vaccines (DTaP for children, Tdap for adolescents and adults), is highly effective in preventing the disease. However, diphtheria has not been eradicated, and recent outbreaks have been reported in Bangladesh, Haiti, and South Africa^25,26^. Additionally, the number of cases worldwide has been increasing in recent years, leading to *C. diphtheriae* being considered a re-emerging pathogen^26^. Understanding diphtheria toxin’s action is crucial for managing and preventing this serious illness.

Anthrax, caused by *Bacillus anthracis*, is highly virulent due to its toxins: protective antigen (PA), lethal factor (LF), and edema factor (EF)^27,28^. PA binds to host cell receptors TEM8 or CMG2, is cleaved into PA63, and forms complexes with LF and EF^27^. These complexes are internalized via endocytosis. Acidification of the endosome triggers PA63 to translocate LF and EF into the cytosol^29–32^. LF cleaves MAPKKs, disrupting cell signaling and causing immune cell death, leading to shock and organ failure. EF increases cAMP levels, disrupting water balance and immune cell function, causing edema. Together, these toxins impair immune responses, aiding bacterial proliferation^28,33–35^. Clinically, anthrax affects humans through cutaneous, inhalational, gastrointestinal, and injection routes, with inhalational anthrax being the most severe. Rapid diagnosis and treatment are essential, highlighting the importance of public health measures, especially in regions with frequent outbreaks. Anthrax therapy involves antibiotics like ciprofloxacin and doxycycline, with early treatment crucial. Antitoxins such as raxibacumab help neutralize toxins, and severe cases may require supportive care^36^. Vaccination, primarily with BioThrax (AVA), is recommended for high-risk groups like military personnel and lab workers. Research is ongoing to create more effective vaccines with fewer doses and longer-lasting immunity. Anthrax’s potential as a bioterrorism agent underscores its relevance in biodefense^37^. The 2001 anthrax attacks in the U.S. prompted efforts to improve detection, prevention, and response strategies, including vaccines, antibiotics, and diagnostic tools.

Recently we identified human alpha-1 antitrypsin (α_1_AT) as an inhibitor of *Bordetella pertussis* toxin (PT) (Lietz et al., under revision). PT also belongs to the group of ADP-ribosylating AB-toxins and is a key virulence factor in causing the severe childhood disease whooping cough^38,39^. α_1_AT was identified by screening of a peptide/protein library derived from human hemofiltrate. α_1_AT is an essential liver-produced protein that protects the lungs from enzyme-induced damage, particularly from neutrophil elastase^40,41^. A genetic disorder, α_1_AT deficiency, results in unregulated tissue degradation in the lower respiratory tract and necessitates intravenous α_1_AT-replacement therapy^42–45^. To treat this deficiency, purified α_1_AT from donor blood, such as Prolastin, is used as an approved medication^44,46,47^. Searching for endogenous human peptides/proteins as therapeutics offers significant advantages. These peptides/proteins are biocompatible and less likely to trigger immune responses, as they naturally occur in the human body^48^. They exhibit high specificity and potency, reducing side effects and improving patient safety. Their ability to regulate various physiological processes allows them to address a wide range of diseases^48,49^. Additionally, drug repurposing of these peptide/protein therapeutics can expedite the development process, lower costs, and utilize existing safety profiles, making it quicker and more economical to bring new treatments to market. In this study, we investigated whether α_1_AT inhibits other AB-type toxins. Our findings revealed that α_1_AT protects cells from C2 toxin, DT, and anthrax fusion toxin intoxication, but not from intoxication with *Clostridioides (C.) difficile* TcdA/TcdB or CDT toxins.

## Results

### *α*_*1*_*AT inhibits intoxication of HeLa cells with C2 toxin*

**Fig. 1:**
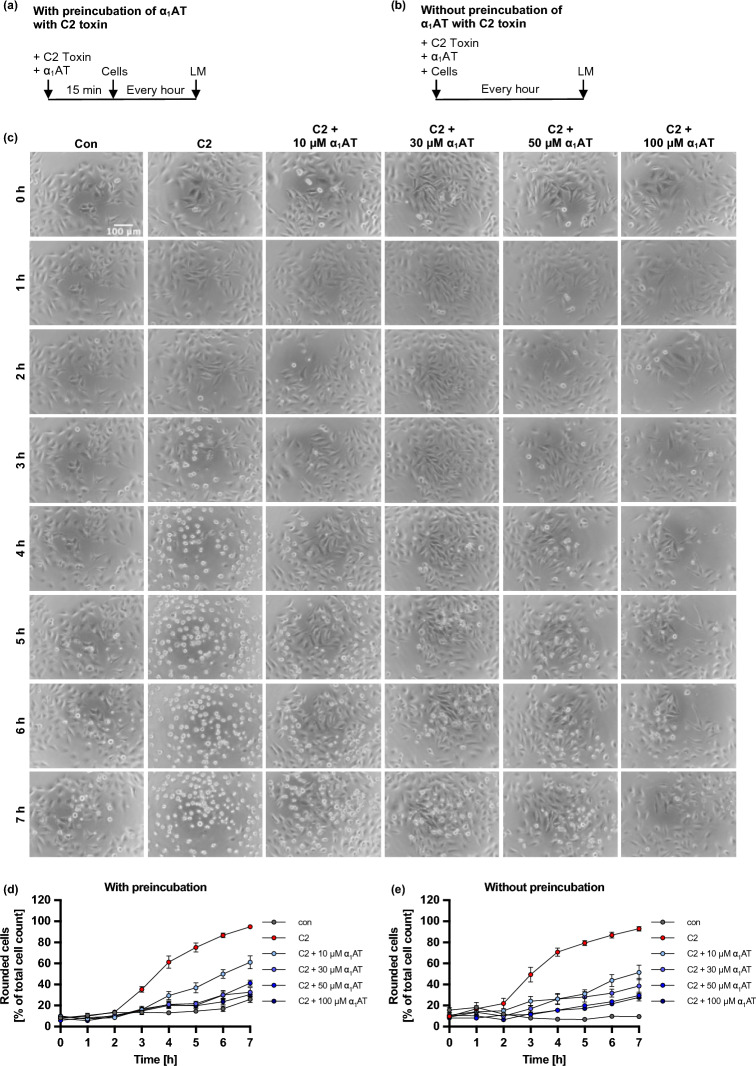
Effect of α_1_AT on C2-toxin mediated cell rounding of HeLa cells. (**a**, **b**) Schematic representation of experimental setup for the cell morphology assay. C2 toxin and α_1_AT were either preincubated for 15 min before addition to cells (**a**) or added simultaneously (**b**). Then, cells were incubated for 7 h at 37 °C, and pictures were taken every hour using the light microscope (LM). (**c**–**e**) Different concentrations of α_1_AT or the respective amount of its solvent (H_2_O) were preincubated for 15 min at RT with C2 toxin (C2 toxin = C2I/C2IIa: 100/200 ng/ml) in FCS-free medium before addition to HeLa cells (**c**, **d**) or added directly (**e**). The cells were incubated for 7 h at 37 °C, and pictures were taken every hour. Rounded cells are given as percent of the total cell count, mean + /− SEM (at least n = 6 and at most n = 9 values from three independent experiments). (**c**) Representative pictures are shown for a representative experiment where C2 toxin was preincubated for 15 min with different concentrations of α_1_AT before addition to HeLa cells.

After identifying α_1_AT as an inhibitor of PT, we explored whether this protective effect could be extended to other AB-type toxins as well and tested C2 toxin, the prototype of clostridial binary toxins (Fig. 1). Since C2 toxin exhibits its enzymatic activity on the key component of the cytoskeleton, G-actin, by ADP-ribosylation, the cytoskeleton collapses which leads to cell rounding, and cell death. The toxin-caused cell rounding and change of morphology was investigated as a specific endpoint for C2 intoxication of HeLa cells. When HeLa cells were intoxicated with C2 toxin in the presence of different concentrations of α_1_AT, a concentration-dependent decrease of cell rounding was observed, indicating inhibition of C2 intoxication by α_1_AT. This effect was more pronounced, when C2 toxin was preincubated with α_1_AT for 15 min at room temperature before addition to HeLa cells (Supplementary Fig. 1). To unravel the mechanism of inhibition by α_1_AT on C2 intoxication, experiments were conducted to investigate the steps of C2 toxin uptake and mode of action in an isolated manner.

### *α*_*1*_*AT inhibits C2 toxin mediated destruction of actin cytoskeleton in a concentration-dependent manner*

**Fig. 2:**
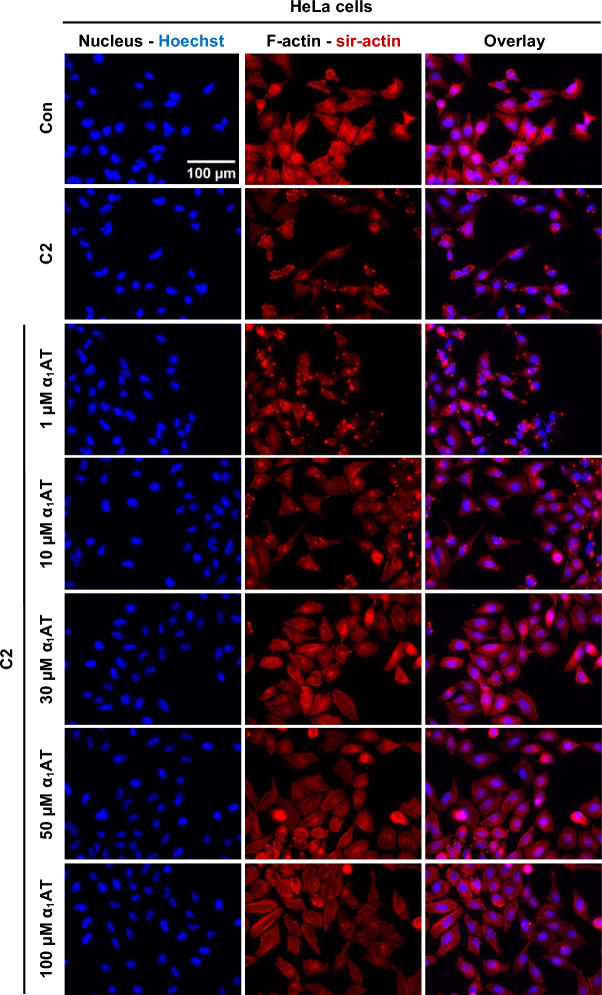
Effect of α_1_AT on F-actin during intoxication of HeLa cells with C2 toxin. C2 toxin (C2I/C2IIa: 100/200 ng/ml) and different concentrations α_1_AT or the respective amount of solvent (H_2_O) were added directly to HeLa cells and incubated for 4 h at 37 °C. Cells were left untreated as control (Con). Subsequently, the cells were washed, fixed, permeabilized, and quenching was performed. Blocking was performed and F-actin was stained using sir-actin (red), and nuclei were stained using Hoechst (blue). Representative images are shown from three independent experiments (n = 3).

To further confirm that α_1_AT inhibits C2 toxin intoxication, fluorescence microscopy experiments were performed analyzing the actin cytoskeleton. Therefore, HeLa cells were intoxicated with C2 toxin for 4 h in the presence or absence of different concentrations of α_1_AT, while F-actin was stained (Fig. 2). Untreated control cells were flat and adherent with an organized cytoskeleton and strong signal for F-actin. In contrast, cells treated with C2 toxin showed dot like signals for F-actin, indicating ADP-ribosylation of G-actin with the resulting destruction of F-actin. At the same time the cells exhibited a round appearance further indicating intoxication. When cells were treated with increasing concentrations of α_1_AT, the signal for F-actin is comparable with the signal for F-actin in untreated control cells. This protective effect was concentration-dependent with 50 µM and 100 µM α_1_AT showing the strongest effect. When HeLa cells were only treated with 100 µM α_1_AT or when cells were treated with 100 µM α_1_AT but not permeabilized, the signal for membrane-permeant F-actin was not altered (Supplementary Fig. 2).

### *α*_*1*_*AT inhibits the enzyme activity of C2I*

**Fig. 3:**
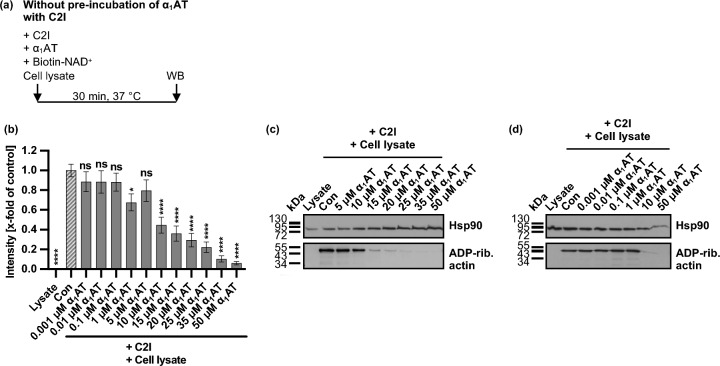
Effect of α_1_AT on enzyme activity of C2I in vitro. (**a**) Schematic representation of experimental setup for the enzyme activity assay. C2I and α_1_AT were directly added to HeLa cell lysate and biotin-NAD^+^, and incubated for 30 min at 37 °C. (**b**–**d**) C2I (20 fmol = 0.001 µM) and different concentrations α_1_AT or the respective amount of solvent (H_2_O) (Con) were added directly to HeLa cell lysate and biotin-NAD^+^ and incubated for 30 min at 37 °C. Cell lysate was left untreated with biotin-NAD^+^ as further control (Lysate). G-Actin which was ADP-ribosylated and biotin-labeled via the incubation with C2I and biotin-labeled NAD^+^ was detected via Western Blot (WB), whereas Hsp90 or Ponceau-S staining served as control for equal protein loading. The bar graph (**b**) shows the quantifications of Western Blot signals from nine independent experiments, while (**c**, **d**) show blots of representative experiments. The intensity values of the bar graph are given as x-fold of the control (con), normalized to Hsp90 or Ponceau-S staining, mean + /− SEM (at least n = 4 at most n = 16 values from nine independent experiments). (**b**) Significance was tested using one-way ANOVA followed by Dunnett’s multiple comparison test and refers to untreated controls (con) (* *p* < 0.1, ** *p* < 0.01, *** *p* < 0.001, **** *p* < 0.0001, ns not significant).

Since the modification of actin was inhibited in α_1_AT treated C2 intoxicated HeLa cells, the effect of α_1_AT on the enzyme activity of C2I in vitro was investigated. Here, cell lysate from HeLa cells was used as source for G-actin. As such, C2I, different concentrations of α_1_AT, cell lysate, and biotin-labeled NAD^+^ were incubated together (Fig. 3, Supplementary Fig. 7). G-Actin which was ADP-ribosylated and biotin-labeled via the incubation with C2I and biotin-labeled NAD^+^, was detected via Western Blot. Interestingly, α_1_AT inhibited the enzyme activity of C2I in vitro, in a concentration-dependent manner. Here, 50 µM α_1_AT already showed a complete inhibition of enzyme activity of C2I in vitro.

### *α*_*1*_*AT inhibits binding of labeled C2 to HeLa cells*

**Fig. 4:**
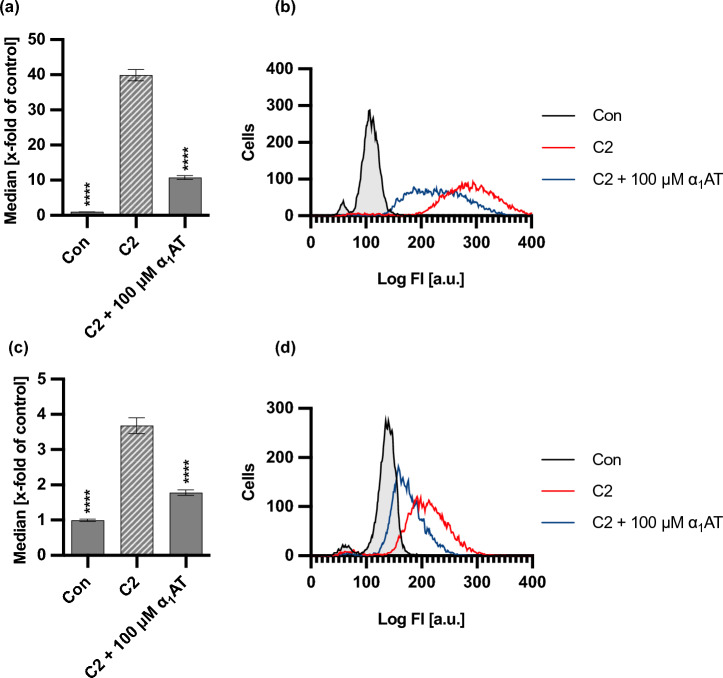
Effect of α_1_AT on binding of labeled C2 toxin to HeLa cells. (**a**–**d**) 488-labeled C2IIa (800 ng/ml) and 405-labeled C2I (800 ng/ml) and 100 µM of α_1_AT or the respective amount of solvent (H_2_O) were added directly to HeLa cells and incubated for 15 min at 4 °C to enable binding of C2 toxin to cells but no internalization. Cells were left untreated (PBS controls from labeling process) as control. After that, cells were washed by centrifugation and 488-labeled C2IIa (**a**) and 405-labeled C2I (**c**) bound to cell surfaces was measured using flow cytometry. Values of median are given as x-fold of the untreated control (Con), mean + /− SEM (n = 9 from three independent experiments). (**b**, **d**) Histograms show fluorescence intensity of cells for one representative experiment. (**a**–**d**) Significance was tested using one-way ANOVA followed by Dunnett’s multiple comparison test and refers to toxin-treated controls (* *p* < 0.1, ** *p* < 0.01, *** *p* < 0.001, **** *p* < 0.0001, ns not significant).

To further unravel the inhibition mechanism of α_1_AT on C2 intoxication, the binding of C2 toxin to cells was analyzed. Therefore, a flow cytometry based binding assay with fluorescently labeled C2I and C2IIa was performed. Here, the labeled toxin components were preincubated with 100 µM α_1_AT for 15 min at room temperature. The approaches were subsequently added to HeLa cells and incubated for further 15 min at 4 °C to enable toxin binding but not internalization. As such, a significantly reduced fluorescence intensity was observed in the presence of α_1_AT, regardless of whether the enzyme component C2I or the binding component C2IIa was fluorescently labeled and detected by flow cytometry (Fig. 4).

### *α*_*1*_*AT reduces detectable C2IIa signal in HeLa cells and inhibits binding of C2 toxin to HeLa cells in a concentration-dependent manner*

**Fig. 5:**
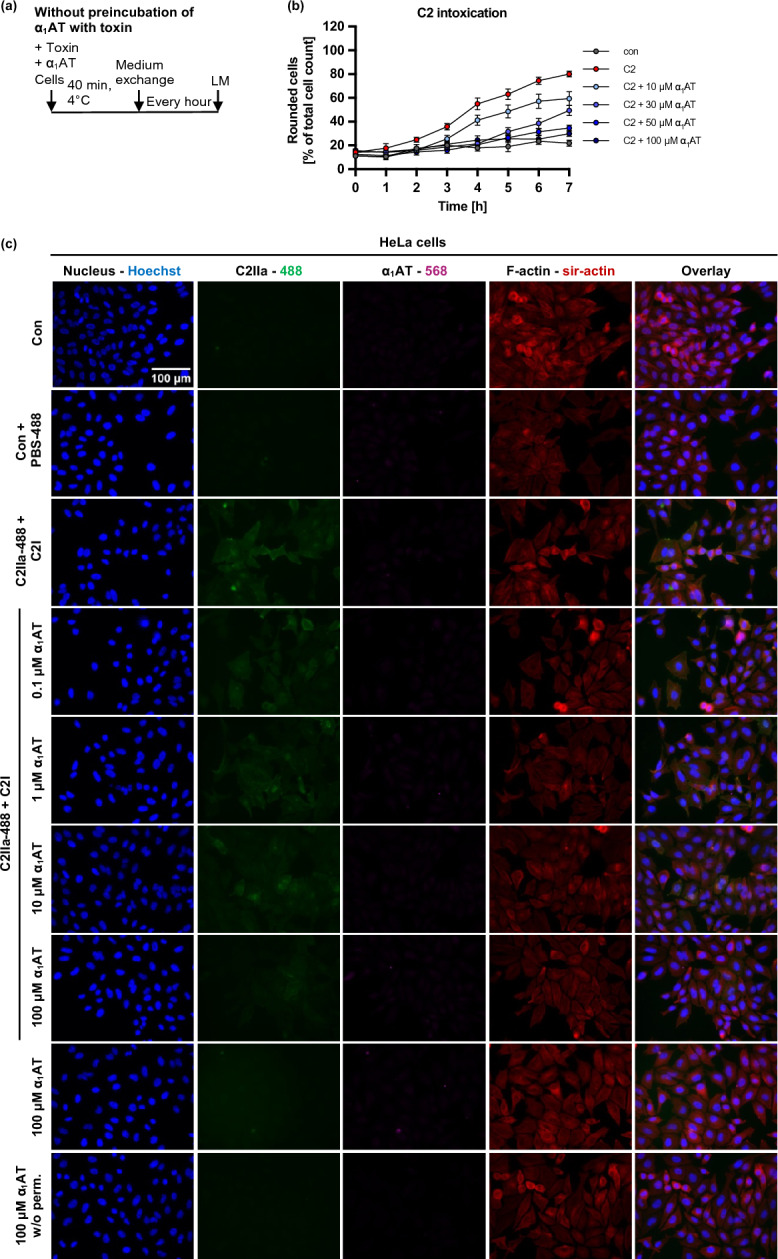
Effect of α_1_AT on binding of C2 toxin to HeLa cells. (**a**) Schematic representation of experimental setup for the cell morphology binding assay. The toxin and α_1_AT were added directly to the cells and were incubated for 40 min at 4 °C. Then, medium exchange to FCS-free medium was performed. The cells were incubated for 7 h at 37 °C, and pictures were taken every hour using the light microscope. (**b**) C2 toxin (C2I/C2IIa: 100/200 ng/ml) and different concentrations of α_1_AT or the respective amount of solvent (H_2_O) were added in FCS-free medium to HeLa cells and incubated for 40 min at 4 °C to enable C2 binding but not internalization. After that medium exchange to FCS-free medium was performed. The cells were incubated for 7 h at 37 °C, and pictures were taken every hour. Rounded cells are given as percent of the total cell count, mean + /− SEM (for each time point n = 9 values from three independent experiments). (**c**) C2 toxin (C2IIa-488/C2I: 33.2/20 nM) and different concentrations α_1_AT or the respective amount of solvent (H_2_O) were mixed, centrifuged, and supernatants were added directly to HeLa cells and incubated for 40 min at 4 °C to enable C2 binding but not internalization. Cells were left untreated as control (Con) or treated with respective amount of PBS-488 (control from toxin labeling) and solvent (H_2_O) (Con + PBS-488). Subsequently, the cells were washed, fixed, permeabilized (as indicated), and quenching was performed. Blocking was performed, and the cells were incubated with a primary antibody for α_1_AT (purple). Primary antibody was detected via a fluorescently labeled secondary antibody, F-actin was stained using sir-actin (red), and nuclei were stained using Hoechst (blue). Representative images are shown from three independent experiments (n = 3).

Furthermore, the inhibition of C2 toxin cell binding by α_1_AT was confirmed by monitoring the cell morphology. Therefore, HeLa cells were incubated with α_1_AT and C2 toxin at 4 °C. After medium exchange, cells were transferred to 37 °C, and cell morphology was monitored (Fig. 5a,b). By this, only C2 toxin that has bound to cells during the incubation at 4 °C leads to intoxication and therefore cell rounding. Here, 100 µM α_1_AT almost completely inhibited C2 intoxication by inhibiting C2 binding to HeLa cells. Moreover, binding experiments based on fluorescence microscopy were conducted using labeled C2IIa. This analysis demonstrated as well that C2 toxin binding is reduced by α_1_AT (Fig. 5c). To investigate whether inhibition of C2 binding by α_1_AT is directed due to C2-α_1_AT precipitate formation or cell membrane-α_1_AT interaction a precipitation analysis (Supplementary Fig. 3, Supplementary Fig. 8) and an assay monitoring cell rounding were conducted (Supplementary Fig. 4) respectively. For the precipitation analysis, TcdB and α_1_AT or α-Defensin 6 as a positive control or C2 toxin with different concentrations of α_1_AT were incubated for 30 min at 37 °C. After that the samples were centrifuged and supernatant and pellet fraction were analyzed using Western Blot. Here, TcdB and C2II/C2IIa were detected. As shown in Supplementary Fig. 3b TcdB does not precipitate with α_1_AT, since most of the TcdB signal is observed in the supernatant fraction. As previously reported^50^, TcdB and α-Defensin 6 form precipitates as the majority of the signal is detected in the pellet fraction. For all the approaches with C2 and α_1_AT in Supplementary Fig. 3c, most of the signal is observed in the supernatant fraction. As such, no concentration-dependent precipitate formation was detected. To test whether α_1_AT inhibits C2 binding by blocking binding sites on the cell surface, a cell morphology assay was conducted where α_1_AT binding to cells was allowed for 40 min at 4 °C. After that, a washing step was performed to remove unbound α_1_AT and the cells were intoxicated with C2 toxin at 37 °C. Then cell rounding was monitored. As shown in Supplementary Fig. 4, pre-incubation of cells with α_1_AT could not inhibit intoxication of cells with C2 toxin. This suggests that presence of α_1_AT during C2 intoxication is required for sufficient toxin inhibition.

**Fig. 6:**
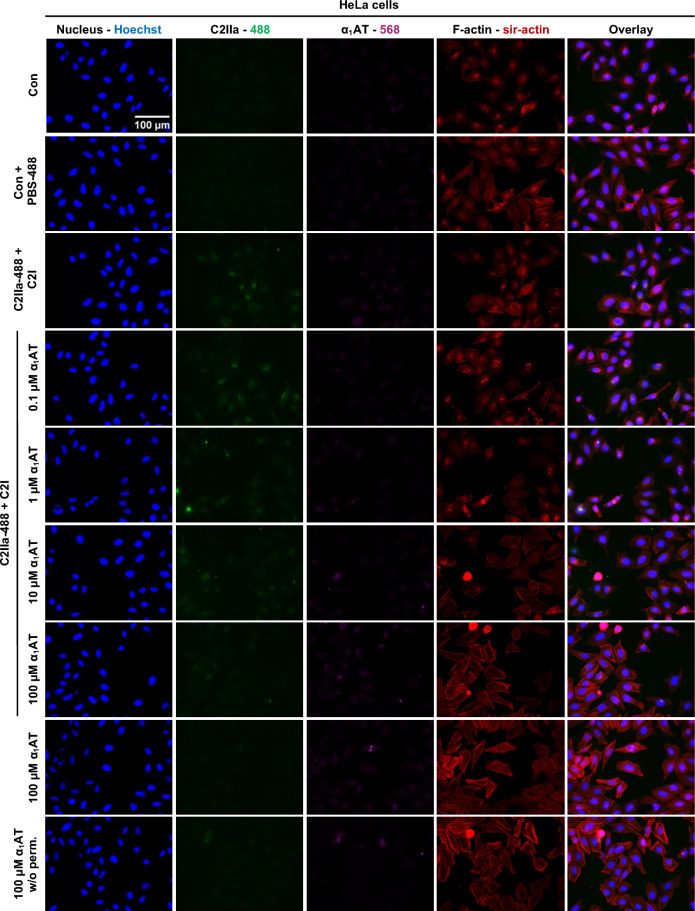
Effect of α_1_AT on endocytosed C2IIa-488 signal in HeLa cells. C2 toxin (C2IIa-488/C2I: 33.2/20 nM) and different concentrations α_1_AT or the respective amount of solvent (H_2_O) were mixed, centrifuged, and supernatants were added directly to HeLa cells and incubated for 30 min at 37 °C. Cells were left untreated as control (Con) or treated with respective amounts of PBS-488 (control from toxin labeling) and solvent (H_2_O) (Con + PBS-488). Subsequently, the cells were washed, fixed, permeabilized (as indicated), and quenching was performed. Blocking was performed, and the cells were incubated with a primary antibody for α_1_AT (purple). Primary antibody was detected via a fluorescently labeled secondary antibody, F-actin was stained using sir-actin (red), and nuclei were stained using Hoechst (blue). Labelled C2IIa is shown in green. Representative images are shown from three independent experiments (n = 3).

Since C2 binding was inhibited by α_1_AT, a fluorescence microscopy experiment was conducted to investigate the endocytosis of C2 in presence of α_1_AT. Therefore, once again fluorescently labeled C2IIa was used. For the investigation of endocytosis, cells were treated with C2 toxin and different concentrations of α_1_AT for 40 min at 37 °C. As such, due to inhibition of C2 binding to HeLa cells also less endocytosed C2 toxin was observed (Fig. 6).

### *α*_*1*_*AT inhibits other bacterial AB-type toxins in a concentration-dependent manner*

**Fig. 7:**
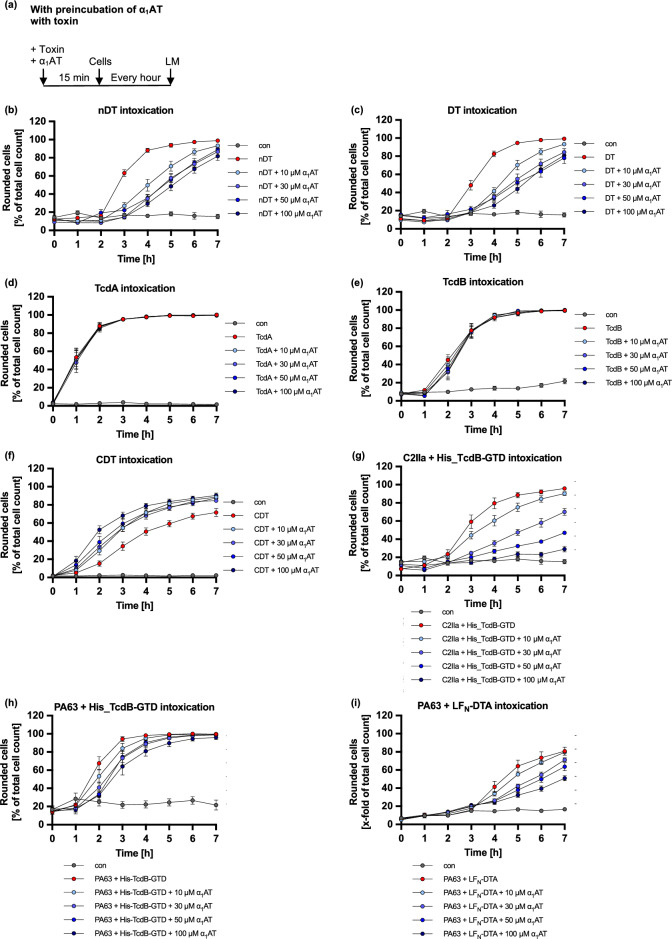
Effect of α_1_AT on other bacterial AB-toxins. (**a**) Schematic representation of experimental setup for the cell morphology assay. The toxins and α_1_AT were preincubated for 15 min before addition to cells. The cells were incubated for 7 h at 37 °C, and pictures were taken every hour using the light microscope (LM). (**b**–**i**) Different concentrations of α_1_AT or the respective amount of solvent (H_2_O) were preincubated for 15 min at RT with the respective toxin in FCS-free medium before addition to HeLa (**b**, **c**, **e**, **f**, **h**, **i**) or Vero (**d**, **f**) cells. Toxin concentrations: nDT: 800 ng/ml, DT 5 nM, TcdA: 180 pM, TcdB: 10 pM, CDT = His_CDTa/CDTb: 5/2 nM, C2IIa + His_TcdB-GTD: 30 nM + 150 nM, PA63 + His_TcdB-GTD: 10 nM + 50 nM, PA63 + LF_N_-DTA: 0.5 nM + 0.25 nM. The cells were incubated for 7 h at 37 °C, and pictures were taken every hour. Rounded cells are given as percent of the total cell count, mean + /− SEM (at least n = 7 and at most n = 12 from three to four independent experiments).

To further investigate the inhibitory capacity of α_1_AT, other bacterial AB-type toxins were tested using an assay monitoring the cell morphology (Fig. 7, Supplementary Fig. 5, and Table 1). Moreover, we recently have published that α_1_AT inhibits binding of PT from *Bordetella pertussis* due to direct interaction of the PT binding subunit with α_1_AT.

**Table 1:** Summary on tested toxins for α_1_AT.

Toxin	Bacterial origin	Toxin structure and function	Intoxication route	Molecular target	Inhibited by α_1_AT	Mechanism of inhibition by α_1_AT
PT	*Bordetella pertussis*	AB_5_ holotoxin, Mono-ADP-ribosyltransferase	Long trip	Gαi	Yes	Inhibition of binding (Lietz et al., under revision)
DT	*Corynebacterium diphtheriae*	Single-chain toxin, Mono-ADP-ribosyltransferase	Short trip	Cytosolic elongation factor 2 (EF-2)	Yes	Inhibition of intoxication but inhibition mechanism not tested yet
TcdA	*Clostridioides difficile*	Single-chain toxin, Monoglucosyltransferase	Short trip	Small Rho-GTPases	No	–
TcdB	*Clostridioides difficile*	Single-chain toxin, Monoglucosyltransferase	Short trip	Small Rho-GTPases	No	–
C2	*Clostridium botulinum*	Binary toxin, Mono-ADP-ribosyltransferase	Short trip	G-actin	Yes	Inhibition of binding and inhibition of enzyme activity
CDT	*Clostridioides difficile*	Binary toxin, Mono-ADP-ribosyltransferase	Short trip	G-actin	No	–
Anthrax fusion toxin PA63 + LF_N_DTA	*Bacillus anthracis, Corynebacterium diphtheriae*	Binary fusion toxin, Mono-ADP-ribosyltransferase	Short trip	Cytosolic elongation factor 2 (EF-2)	Yes	Inhibition of binding subunit PA63

Seven toxins were tested for inhibition of α_1_AT. Summarized are the bacterial origin, toxin structure and function, the intoxication route (short trip: enzyme domains translocate from endosomes into the cytosol; long trip: retrograde trafficking from toxin through endosomes, Golgi apparatus, and ER, enzyme domains translocate from the ER into the cytosol), their molecular target, and the mechanism of toxin inhibition by α_1_AT.

Diphtheria toxin (DT) is a single-chain ADP-ribosylating toxin that is taken up into cells by receptor-mediated endocytosis and translocates its enzyme domain from early endosomes into the cytosol to modify elongation factor 2. In HeLa cells, intoxication with DT leads to cell rounding^51^. DT requires proteolytic activation by the membrane-anchored protease furin to enter cells. However, DT as well as proteolytically activated DT, nicked DT (nDT), were both inhibited by α_1_AT in a comparable manner (Fig. 7b,c). This indicates that inhibition of DT by α_1_AT might also be independent of α_1_AT’s ability to inhibit serine proteases or other proteases such as furin. Flow cytometry analysis revealed that α_1_AT did not hinder the binding of an enzymatically inactive, eGFP-fused DT variant (His-eGFP-CRM197) to cells (Supplementary Fig. 6), suggesting a distinct inhibition mechanism compared to that of C2 toxin and PT.

Further single-chain AB-toxins translocating their enzyme domains from endosomes to the cytosol are *C. difficile* toxins TcdA and TcdB. In contrast to DT and PT, TcdA and TcdB have glucosyltransferase activity targeting small Rho GTPases. This leads also to cell rounding. No inhibition of α_1_AT on intoxication with TcdA or TcdB was observed (Fig. 7d,e).

Next to single-chain and AB_5_-type toxins, binary toxins show a distinct structure harboring their A- and B-domains on two separate proteins, then called components. *Clostridium (C.) botulinum* C2 toxin and *C. difficile* CDT are binary toxins that both translocate their enzyme components from early endosomes and ADP-ribosylate G-actin in the cytosol. This also leads to rounding of adherent cells. Interestingly, α_1_AT protected cells from intoxication by C2 toxin but not by CDT (Fig. 7f).

These results together with the finding that α_1_AT inhibits the binding of PT to cells, prompted us to test different combinations of binding/transport and enzyme domains of toxins. First, we tested whether the transport of the enzyme domain of TcdB, His_TcdB-GTD via the transport component of C2 toxin, C2IIa or the transport component of anthrax toxin, protective antigen (PA63), is inhibited by α_1_AT. It was recently shown that C2IIa not only can transport its enzyme component C2I but also His-tagged enzymes into the cytosol of cells^52^. Moreover, transport of His-tagged enzymes was also shown for the transport component of anthrax toxin, PA63^53,54^. Comparable to DT, PA83 of anthrax toxin undergoes proteolytic cleavage by furin, resulting in PA63, which is required for its toxic activity^55^. Figure 7g reveals that α_1_AT inhibits intoxication of cells by the combination of C2IIa and His_TcdB-GTD in a concentration-dependent manner. Figure 7h show inhibition of intoxication with the combination of PA63 and His_TcdB-GTD by α_1_AT. Next, we tested whether the transport of the fusion protein LF_N_-DTA, where LF_N_ mediates the uptake of the enzyme domain DTA of DT into the cytosol, via the transport component of anthrax toxin, PA63, is inhibited by α_1_AT. LF_N_ is the N-terminal part of the enzyme component of anthrax lethal toxin which mediates the interaction with PA63. DTA was fused to LF_N_ to obtain a robust morphological readout (cell rounding). Results in Fig. 7i also show only inhibition by α_1_AT if cells were intoxicated with the combination of PA63 and LF_N_-DTA. Taken together, these results indicate the binding subunit of the toxin most likely determines whether cells are protected from intoxication by α_1_AT.

Taken together, the results underscore that α_1_AT exerts inhibitory effects not only on C2 toxin and PT but also on diverse bacterial AB-type toxins. Notably, the inhibition of toxin binding emerges as a pivotal mechanism, complemented by the modulation of enzyme activity. This multifaceted inhibition suggests the promise of α_1_AT as a therapeutic agent against a spectrum of toxin-mediated diseases, proposing a versatile strategy for treating multiple pathologies rather than targeting individual afflictions exclusively.

## Discussion

In this study, we explored the inhibitory effects of α_1_AT on the C2 toxin produced by *Clostridium botulinum*, diphtheria toxin, and an anthrax fusion toxin. Our findings demonstrate that α_1_AT, a serine protease inhibitor identified from a hemofiltrate library screen for inhibitors of PT from *Bordetella pertussis*, is a multitoxin inhibitor.

α_1_AT is essential for regulating protease activity and controlling inflammation. It protects lung tissue from damage caused by enzymes like neutrophil elastase, released by immune cells^42^. In healthy individuals, α_1_AT plasma levels range from 0.9 to 2 mg/ml (approximately 17–38 μM). During acute inflammation, these levels can increase four to five times, highlighting α_1_AT’s role as an acute phase protein in preventing inflammation-induced tissue damage^40^. Interestingly, we have observed that in *Bordetella Pertussis* infected mice mRNA levels of the murine genes of α_1_AT, Serpin1a-e, were downregulated (Lietz et al., under revision). This suggests that during bacterial infection with *Bordetella Pertussis* α_1_AT levels might be reduced, ameliorating lung tissue damage, and toxin function due to protease imbalance in favor of neutrophil elastase. In the lungs, physiological α_1_AT concentrations range from 10 to 40 μM in alveolar interstitial fluid and about 2–5 μM in alveolar extracellular lining fluid^43,47,56^. Those concentrations have shown to be protective in our cell-based experiments but if α_1_AT levels are reduced protection against bacterial toxins might be lost. Analyzing serum levels of α_1_AT in infected patients would give further valuable insights. α_1_AT products like Prolastin, derived from purified donated blood, have been used to treat genetic α_1_AT deficiency for decades^44^. These products are typically given intravenously to address chronic tissue degradation in the lower respiratory tract due to the deficiency. Alternatively, α_1_AT can be administered via inhalation, enabling much higher doses than standard treatments^41^. Doses as high as 250 mg/kg have shown a fivefold increase in serpin concentration within the lung’s epithelial lining fluid without causing adverse effects^47^.

Our study found that α_1_AT protects cells from intoxication with C2 toxin, DT, and anthrax fusion toxin starting at concentrations around 10–30 μM, which are within the physiological range. The protective effect was more pronounced at higher concentrations, with the most significant impact observed at concentrations up to 100 μM. Interestingly, comparable concentrations have been previously found to inhibit PT (Lietz et al., under revision). The identification of α_1_AT as an inhibitor of multiple clinically relevant toxins, including C2 toxin, DT, anthrax toxin, and PT highlights its potential for broad-spectrum anti-toxin therapy. The repurposing of α_1_AT could provide a rapid and effective treatment option for various toxin-mediated illnesses, leveraging its established clinical use and safety profile.

Specifically, α_1_AT inhibits the intoxication of cells by C2 toxin through two distinct mechanisms: inhibition of C2 toxin binding to cells and inhibition of the enzyme activity of the C2I component. This dual mechanism of action enhances its efficacy as an inhibitor. Inhibition of binding is most likely directed via the interaction of C2 toxin and α_1_AT, since pre-incubation of cells with α_1_AT was not sufficient for inhibition of C2 intoxication. However, no precipitate formation was observed, suggesting that C2 toxin and α_1_AT do not from aggregates. Moreover, our study found that α_1_AT inhibits both C2 toxin and anthrax fusion toxin, as well as the enzyme domain His_TcdB-GTD transported by PA63, the binding component of anthrax toxin. Moreover, also DT and nicked DT were inhibited by α_1_AT. As a common feature many bacterial AB-type toxins require activation via furin cleavage. Taken our results together, inhibition of furin by α_1_AT might be part of the inhibitory mechanism of α_1_AT, but inhibition of these toxins does not solely rely on inhibition of furin, since also activated toxins (nicked DT and PA63) and toxins that are not dependent on furin cleavage are inhibited. However, others have shown that an engineered α_1_AT variant, α_1_AT Portland, designed as a furin inhibitor can be employed as an antipathogenic agent that can be used prophylactically to block furin-dependent cell killing by *Pseudomonas* exotoxin A and is able to form SDS- and heat-stable serpin/proteinase complexes^57,58^. As such, furin inhibition by α_1_AT plays a neglectable role in the context of our study. Moreover, previous results (Lietz et al., under revision) have shown that another member of the serpin-family, antithrombin, was not able to inhibit PT, showing that protease inhibition activity is not required for toxin inhibition and that basis of inhibitory capacity of α_1_AT probably lies within unique characteristics of structure or amino acid composition.

In contrast, α_1_AT did not inhibit TcdA, TcdB or CDT toxin. All five toxins use different cell surface receptors^7,27,59,60^. C2 toxin, anthrax toxin and CDT belong to the group of binary toxins and share between approximately 30–40% sequence homology in their activated B-components^61^. However, it was recently shown that the crystal structure of CDTb differs from the PA structure and an additional receptor-binding domain was discovered in CDTb, which is absent in the protective antigen^62^. This finding opens up new avenues for research into the design of inhibitors that can target structurally similar toxins, potentially broadening the scope of α_1_AT’s therapeutic applications. Further research is needed to fully understand these mechanisms and to explore the potential for α_1_AT to inhibit other similar toxins.

Anthrax toxin, produced by *Bacillus anthracis*, remains a significant concern due to its potential use as a bioterrorism agent and its role in zoonotic infections^37^. The toxin comprises three components: protective antigen (PA; PA63, proteolytically activated), lethal factor (LF), and edema factor (EF)^27^. PA binds to host cell receptors and facilitates the entry of LF and EF into cells, leading to lethal and edema responses, respectively. Here, we investigated the anthrax fusion toxin PA63 + LF_N_DTA consisting of the N-terminal part of LF (LF_N_) that facilitates the interaction with PA63 and the enzyme domain of diphtheria toxin (DTA)^32^. This allows a morphological readout on cultivated HeLa cells due to DTA-induced cell rounding while investigating the effect of α_1_AT on PA63 and LF_N_. α_1_AT inhibited intoxication with PA + LF_N_DTA most likely due to inhibition of PA63-binding to cells. This is supported by the finding that intoxication with His_TcdB-GTD + PA63 is also inhibited by α_1_AT although intoxication with the wildtype TcdB with its original receptor domain is not affected by α_1_AT. The historical impact of anthrax, its high mortality rate when inhaled, and the ease of spore dissemination underscore the need for effective countermeasures. The ability of α_1_AT to inhibit anthrax toxin adds a potential dimension to its therapeutic potential, offering a promising defense against both natural outbreaks and bioterrorism threats. Developing α_1_AT as an inhibitor could provide a rapid, effective response to anthrax exposure, thereby enhancing public health security.

Although a widespread vaccination against diphtheria is available, outbreaks in Bangladesh, Haiti, and South Africa have been reported recently^25,26^. Additionally, the number of cases worldwide has been increasing in recent years, leading to *C. diphtheriae* being considered a re-emerging pathogen^26^. Treatment strategies for diphtheria could benefit from incorporating α_1_AT, particularly since high concentrations can be achieved through inhalation. This method aligns well with the disease’s pathology, as DT is primarily released in the upper respiratory tract, where it causes severe symptoms and airway obstruction^19,63^.

While our study provides compelling evidence for the inhibitory effects of α_1_AT on multiple toxins, it is limited by its in vitro nature using cell culture experimentation. Future studies should include in vivo experiments to confirm these findings and assess the therapeutic potential of α_1_AT in animal models. Additionally, exploring the structure–activity relationship of α_1_AT and its interactions with various toxins could lead to the development of optimized inhibitors. In conclusion, α_1_AT shows promise as a multitoxin inhibitor with potential applications in treating toxin-mediated diseases. Its established use in treating α_1_AT deficiency, coupled with its broad-spectrum inhibitory effects, supports its repurposing as an anti-toxin therapeutic. Further research and in vivo studies are necessary to fully realize its potential in clinical settings.

## Methods

### Compounds and reagents

The native toxins TcdA and TcdB from *C. difficile* VPI 10,463 were expressed and purified as described elsewhere^64^. C2I and C2IIa were purified according to^65^. His_TcdB-GTD was purified as described earlier^52^. DT was purchased from Calbiochem/Merck KGaA (Bad Soden/Darmstadt, Germany). DT was activated in vitro by trypsin digestion as described before^51^, resulting in nicked DT (nDT). His-eGFP-CRM197 was expressed and purified as described before^66^, as well as CDTa and CDTb^67^ and PA63^52^. The fusion toxin LF_N_-DTA was expressed and purified as described earlier^32^. As α_1_AT source the commercially available drug Prolastin^®^ purchased from Grifols (Frankfurt am Main, Germany) was used. The peptide α-defensin-6 was purchased from PeptaNova (Sandhausen, Germany).

### Cell lines

Unless mentioned differently, the materials for the cultivation of all cell lines were purchased from Gibco (Thermo Fisher Scientific, Waltham, MA, USA). The used cell lines included Vero cells (African green monkey kidney cells; DSMZ, Braunschweig, Germany), and HeLa cells (cervical carcinoma cells; DSMZ, Braunschweig, Germany). Vero cells and HeLa cells were cultivated in MEM, while MEM was supplemented with 10% FCS, 1 mM sodium pyruvate, 0.1 mM non-essential amino acids and 100 U/ml penicillin and 100 g/ml streptomycin. Cells were cultivated under humidified conditions at 37 °C with 5% CO_2_ and trypsinized and reseeded every two to three days for at most 25 times. For intoxication experiments, cells were seeded in culture dishes one or two days before and treated in FCS-free media with toxins and the respective compounds.

### Cell morphology assays

For the analysis of cell morphological changes, cells were seeded in 96-well plates one or two days prior to treatment with toxins, α_1_AT, and water (solvent control). Before the addition of the components to cells, α_1_AT or water (solvent control) was preincubated with the respective toxin for 15 min at room temperature in FCS-free medium. The morphology of cells was documented using light microscopy every hour for at least 6 h using 20 × magnification a Leica DMi1 microscope connected to a Leica MC170 HD camera (both Leica Microsystems GmbH, Wetzlar, Germany). Rounded cells were counted from complete pictures taken, using the online software Neuralab.

### Enzyme activity assay of C2I from cell lysates

For the analysis of the in vitro enzyme activity of C2I, cell lysate from HeLa cells was generated. As such, HeLa cells were seeded in 10 cm culture dishes and grown for two to three days. After that, the cells were washed, and frozen for lysis. Next, ADP-ribosylation buffer 20 mM Tris–HCl, 1 mM EDTA, 1 mM DTT, 5 mM MgCl2, 1:50 freshly added cOmplete^™^ (Roche); pH 7.5) was added, and the cell lysate was collected in tubes. The cell lysate was centrifuged at 10,000 × *g* for 1 min, the supernatant was transferred into a new tube, and the protein concentration was measured using the Nanodrop. Different concentrations of α_1_AT, water (solvent control), and 30 µg HeLa cell lysate were mixed in ADP-ribosylation buffer with a total reaction volume of 20 µl. Subsequently, C2I (20 fmol = 0.001 µM) and biotin-labeled NAD^+^ (1 µM; R&D Systems) were added for labeling of the ADP-ribosylation of actin during the 30 min incubation at 37 °C. After that, the samples were subjected to gel electrophoresis and Western blotting. The ADP-ribosylated actin by C2I was detected using streptavidin-peroxidase (Strep-POD, Sigma-Aldrich, Merck, Darmstadt, Germany). Hsp90 (primary antibody from Santa Cruz Biotechnology, Dallas, TX, USA) served as loading control, and the signal quantification was performed using the ImageJ software v.1.52.a (NIH).

### Gel electrophoresis and western blotting

After the samples were prepared, Laemmli buffer (0.3 M Tris–HCl, 10% SDS, 37.5% glycerol, 0.4 mM bromophenol blue, 100 mM DTT) was added, and the samples were heat-denatured at 95 °C for 10 min. For protein separation via gel electrophoresis, 12.5% acrylamide gels were used. The transfer of proteins from the gels onto nitrocellulose membranes was performed by semi-dry Western blotting and controlled by staining the membranes with Ponceau-S-staining (AppliChem GmbH, Darmstadt, Germany). Then, the membranes were blocked in 5% skim milk powder in PBS-T (PBS containing 0.1% Tween 20) for at least 30 min at room temperature, followed by washing steps in PBS-T. Next, incubation with primary and secondary antibodies in PBS-T separated by washing steps was performed. After the final washing steps, signals were detected using Pierce ECL Western blotting substrate (Thermo Fisher Scientific, Waltham, MA, USA) and X-ray films (AGFA Health Care, Mortsel, Belgium) or the iBright 1500 system (Thermo Fisher Scientific).

### Binding analysis using flow cytometry

For the binding analysis based on flow cytometry, C2I was labeled with DyLight^®^ 405 NHS Ester (Thermo Fisher Scientific, Rockford, IL, USA) and C2IIa was labeled with DyLight^®^ 488 NHS Ester (Thermo Fisher Scientific, Rockford, IL, USA) according to the manufacturer’s protocol using the Zeba^™^ Spin Desalting Columns (7 K MWCO, Thermo Fisher Scientific, Waltham, MA, USA) to remove excess dye. After that, cells were grown in a culture dish, detached using 25 mM EDTA in PBS, and resuspended in PBS. 488-labeled C2IIa and 405-labeled C2I or His_eGFP_CRM197, α_1_AT, and water (solvent control) were preincubated for 15 min at room temperature or added directly to cells (1 or 2 × 10^6^ in 0.2 mL per sample) for 15 min at 4 °C to enable binding of C2 or His_eGFP_CRM197 to cells but no internalization. The samples were washed by centrifugation, and the cell fluorescence was measured at an excitation wavelength of 488 nm or 405 nm using the BD FACS Celesta™ flow cytometer (Becton, Dickinson and Company, Franklin Lakes, NJ, USA) and the BD FACSDiva^™^ software 8.0.1.1. Cell gating and data analysis was performed using Flowing Software v2.5.1. (Turku Centre of Biotechnology, Finland).

### Cellular binding and uptake analysis using fluorescence microscopy

For all immunostaining and fluorescence microscopy experiments, cells were seeded and grown for one day in 18-well µ-slides (ibidi GmbH, Gräfelfing, Germany). Subsequently, the cells were treated with α_1_AT or water (solvent control) and were intoxicated with C2 toxin (C2IIa-488 or C2IIa and C2I) in FCS-free medium for the uptake analysis for 4 h at 37 °C, for the endocytose analysis for 40 min at 37 °C or for the binding analysis for 40 min at 4 °C. Approaches including labeled C2IIa were centrifuged before addition to cells. After that, the cells were washed with PBS, fixed with 4% paraformaldehyde for 20 min, permeabilized using 0.4% (v/v) Triton X-100 in PBS for 5 min if required, and quenching was performed for 2 min in glycine (100 mM in PBS). After that, the cells were blocked for 1 h at 37 °C in PBS-T (PBS containing 0.1% Tween 20) containing 10% normal goat serum (Jackson ImmunoResearch, West Grove, PA, USA) and 1% BSA. The incubation with the primary antibody for α_1_AT (Alpha-1-Antitrypsin antibody, Proteintech, Planegg-Martinsried, Germany) in blocking solution was performed for 1 h at 37 °C. F-actin was stained for 1 h at 37 °C using the membrane-permeant sir-actin (SiR-actin kit, Spirochrome, Stein am Rhein, Switzerland). The primary antibody for α_1_AT was detected via fluorescently labeled secondary antibody in blocking solution for 1 h at 37 °C, and cell nuclei were stained for 5 min using Hoechst 33,342 (1:10,000, Thermo Fisher Scientific, Waltham, MA, USA). After staining, the slides were examined via microscopy using the BZ-X810 Keyence fluorescence microscope (Keyence Deutschland GmbH, Neu-Isenburg, Germany) and BZ-X800Viewer v1.3.0.

### In vitro precipitation assay

For the in vitro precipitation analysis, C2 (C2IIa/C2I: 33.2/20 nM) and TcdB (50 ng) and inhibitors, α_1_AT and α-defensin-6 (6 µM) were incubated for 30 min in PBS at 37 °C. After incubation, the samples were centrifuged for 20 min, 14,000 rpm at 4 °C. Subsequently, the supernatant fraction was separated and transferred into a new tube and the pellet was resuspended in PBS. The samples were subjected to gel electrophoresis and Western blotting. C2 was detected using an antiserum against C2II while TcdB was detected using an anti-TcdB antibody (Anti-Clostridium difficile Toxin B antibody, Abcam, Cambridge, UK).

### Reproducibility of experiments and statistics

All experiments were performed independently from each other at least three times. The number of replicates (n) for experiments or tested conditions is given in the figure legends. Moreover, representative results are shown in the figures and if not stated otherwise in the figure legends, the statistical analysis performed was a one-way ANOVA in combination with Dunnett’s multiple comparison test using GraphPad Prism Version 9 (GraphPad Software Inc., San Diego, CA, USA). The obtained p values are depicted as follows: ns = not significant *p* > 0.05, * *p* < 0.05, ** *p* < 0.01, *** *p* < 0.001, **** *p* < 0.0001.

## Supplementary Information

Supplementary Figures.

## Data Availability

The datasets generated and/or analyzed during the current study are either included in the study or available from the corresponding author on reasonable request.
