# Supplementary material for: Alpha-1 antitrypsin inhibits Clostridium botulinum C2 toxin, Corynebacterium diphtheriae diphtheria toxin and B. anthracis fusion toxin
Source: Sci Rep. 2024 Sep 11;14:21257. doi: 10.1038/s41598-024-71706-7 (PMC11390955; doi:10.1038/s41598-024-71706-7)
Supplement: Supplementary file 1 — Supplementary Figures. [file 41598_2024_71706_MOESM1_ESM.docx]

**Supplementary Figures - Alpha-1 antitrypsin inhibits *Clostridium botulinum* C2 toxin, *Corynebacterium diphtheriae* diphtheria toxin and *B. anthracis* fusion toxin**

**Supplementary Figure 1: Effect of α_1_AT on C2-toxin mediated cell rounding of HeLa cells. (a, b)** Different concentrations of α_1_AT or the respective amount of its solvent (H_2_O) were preincubated for 15 min at RT with C2 toxin (C2 toxin = C2I/C2IIa: 100/200 ng/ml) in FCS-free medium before addition to HeLa cells (a) or added directly (b). The cells were incubated for 7 h at 37 °C, and pictures were taken every hour. Rounded cells are given as percent of the total cell count after 7 h, mean +/- SEM (at least n = 6 and at most n = 9 values from three independent experiments). Significance was tested using one-way ANOVA followed by Dunnett’s multiple comparison test and refers to C2 toxin treated controls (C2) (* p < 0.1, ** p < 0.01, *** p < 0.001, **** p < 0.0001, ns not significant).

**Supplementary Figure 2: Effect of α_1_AT on F-actin during intoxication of HeLa cells with C2 toxin.** C2 toxin (C2I/C2IIa: 100/200 ng/ml) and different concentrations α_1_AT or the respective amount of solvent (H_2_O) were added directly to HeLa cells and incubated for 4 h at 37 °C. Cells were left untreated as control (Con). Subsequently, the cells were washed, fixed, permeabilized, and quenching was performed. Blocking was performed and F-actin was stained using sir-actin (red), and nuclei were stained using Hoechst (blue). Representative images are shown from three independent experiments (n = 3).

**Supplementary Figure 3: Effect of α_1_AT on precipitation with C2 toxin *in vitro*.** **(a)** Schematic representation of experimental setup for the *in vitro* precipitation analysis. C2 toxin and α_1_AT were added to PBS and were incubated for 30 min at 37 °C. Then, the samples were centrifuged, supernatant and pellet were separated, and Western Blot (WB) was performed. **(b)** TcdB (50 ng) and different concentrations α_1_AT or the respective amount of solvent (H_2_O) for controls (con) were mixed in PBS and incubated for 30 min at 37°C. After that samples were centrifuged, supernatant and pellet were separated, and analyzed via Western Blot. TcdB was detected, using an anti-TcdB antibody. **(c)** C2 toxin (C2IIa/C2I: 33.2/20 nM) and different concentrations α_1_AT or the respective amount of solvent (H_2_O) for controls (con) were mixed in PBS and incubated for 30 min at 37°C. After that samples were centrifuged, supernatant and pellet were separated, and analyzed via Western Blot. C2 toxin was detected via C2II antiserum, recognizing C2II and C2IIa. (S = supernatant, P = pellet)

**Supplementary Figure 4: Effect of preincubation with α_1_AT previous to C2-toxin intoxication on cell rounding of HeLa cells. (a)** Schematic representation of experimental setup for the cell morphology assay. α_1_AT was preincubated for 40 min at 4 °C on HeLa to allow binding of α_1_AT to cell membranes. Then, the cells were intoxicated with C2 toxin, incubated for 7 h at 37 °C, and pictures were taken every hour using the light microscope. **(b, c)** Different concentrations of α_1_AT or the respective amount of its solvent (H_2_O) were preincubated for 40 min at 4 °C on HeLa cells and subsequently treated with C2 toxin (C2 toxin = C2I/C2IIa: 100/200 ng/ml) in FCS-free medium. The cells were incubated for 7 h at 37 °C, and pictures were taken every hour. Rounded cells are given as percent of the total cell count, mean +/- SEM for all time points (b) or after 7 h (c) (n = 9 values from three independent experiments). Significance was tested using one-way ANOVA followed by Dunnett’s multiple comparison test and refers to C2 toxin treated controls (C2) (* p < 0.1, ** p < 0.01, *** p < 0.001, **** p < 0.0001, ns not significant).

**Supplementary Figure 5.** **Effect of α_1_AT on bacterial AB-toxins.** Different concentrations of α_1_AT or the respective amount of solvent (H_2_O) were preincubated for 15 min at RT with the respective toxin in FCS-free medium before addition to HeLa or Vero cells. Toxin concentrations: nDT: 800 ng/ml, DT 5 nM, TcdA: 180 pM, TcdB: 10 pM, CDT = His_CDTa/CDTb: 5/2 nM, C2IIa + His_TcdB-GTD: 30 nM + 150 nM, PA63 + His_TcdB-GTD: 10 nM + 50 nM, PA63 +LF_N_-DTA: 0.5 nM + 0.25 nM. The cells were incubated for 7 h at 37 °C, and pictures were taken every hour. Rounded cells are given as percent of the total cell count, mean +/- SEM (at least n = 7 and at most n = 12 from three to four independent experiments). Representative pictures are show after 4 h.

**Supplementary Figure 6: Effect of α_1_AT on binding of enzymatic inactive DT toxins to HeLa cells.** **(a-b)** His_eGFP_CRM197 (250 nM) and different concentrations of α_1_AT or the respective amount of solvent (H_2_O) were either directly added to HeLa cells **(a)** or pre-incubated for 15 min at RT before addition to HeLa cells **(b)**. After that, cells were incubated for 15 min at 4 °C to enable binding of His_eGFP_CRM197 to cells but no internalization. Cells were left untreated as control. After that, cells were washed by centrifugation and His_eGFP_CRM197 bound to cells surfaces was measured using flow cytometry. Values of median are given as x-fold of the untreated control (con), mean +/- SEM (n = 9 values from three independent experiments). Significance was tested using one-way ANOVA followed by Dunnett’s multiple comparison test and refers to toxin treated controls (His_eGFP_CRM197) (* p < 0.1, ** p < 0.01, *** p < 0.001, **** p < 0.0001, ns not significant).


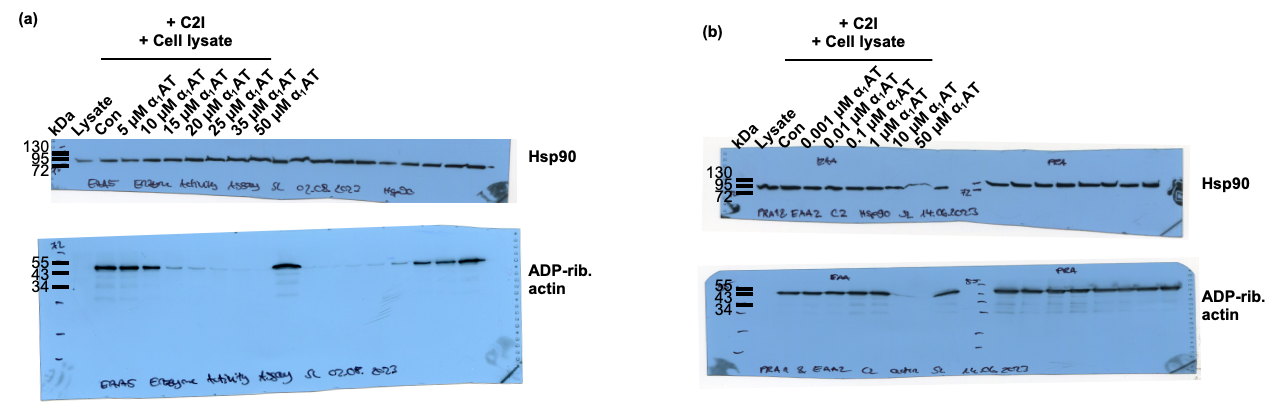


**Supplementary Figure 7: Original uncropped blots corresponding to images from Figure 3 c and d. (a)** Uncropped blot image of Figure 3c. **(b)** Uncropped blot image of Figure 3d.


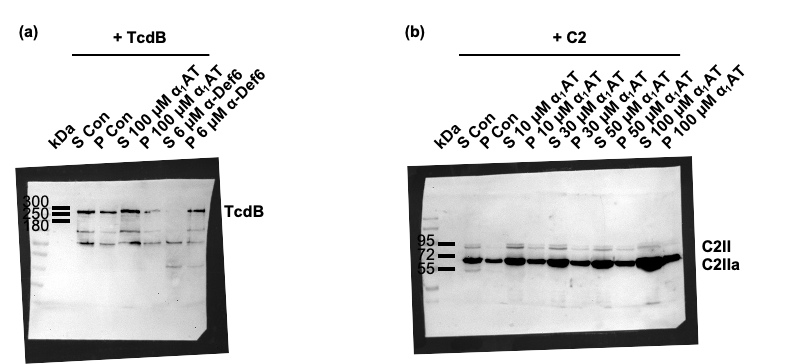


**Supplementary Figure 8: Original uncropped blots corresponding to images from Supplementary Figure 3 b and c. (a)** Uncropped blot image of Supplementary Figure 3b. **(b)** Uncropped blot images of Supplementary Figure 3c.
